# Maternal and Perinatal Outcomes of Pregnancies Complicated by Chronic Hypertension Followed at a Referral Hospital

**DOI:** 10.1055/s-0040-1709190

**Published:** 2020-05

**Authors:** Gabriela Pravatta Rezende, Laura Casagrande, José Paulo Siqueira Guida, Mary Angela Parpinelli, Fernanda Garanhani Surita, Maria Laura Costa

**Affiliations:** 1 Departament of Obstetrics and Gynecology, Universidade de Campinas, Campinas, SP, Brazil

**Keywords:** preeclampsia, chronic hypertension, obstetrics, maternal morbidity, complications of pregnancy, pré-eclâmpsia, hipertensão crônica, obstetrícia, morbidade materna, complicações da gravidez

## Abstract

**Objective** To assess maternal and perinatal outcomes of pregnancies in women with chronic hypertension (CH).

**Methods** Retrospective cohort of women with CH followed at a referral center for a 5 year period (2012–2017). Data were obtained from medical charts review and described as means and frequencies, and a Poisson regression was performed to identify factors independently associated to the occurrence of superimposed pre-eclampsia (sPE).

**Results** A total of 385 women were included in the present study; the majority were > than 30 years old, multiparous, mostly white and obese before pregnancy. One third had pre-eclampsia (PE) in a previous pregnancy and 17% of them had organ damage associated with hypertension, mainly kidney dysfunction. A total of 85% of the patients used aspirin and calcium carbonate for pre-eclampsia prophylaxis and our frequency of sPE was 40%, with an early onset (32.98 ± 6.14 weeks). Of those, 40% had severe features of PE, including 5 cases of HELLP syndrome; however, no cases of eclampsia or maternal death were reported. C-section incidence was high, gestational age at birth was 36 weeks, and nearly a third (115 cases) of newborns had complications at birth One third of the women remained using antihypertensive drugs after pregnancy.

**Conclusion** Chronic hypertension is related with the high occurrence of PE, C-sections, prematurity and neonatal complications. Close surveillance and multidisciplinary care are important for early diagnosis of complications.

## Resumo

**Objetivo** Avaliar os resultados maternos e perinatais em gestação de mulheres com hipertensão crônica.

**Métodos** Coorte retrospectiva de mulheres hipertensas crônicas acompanhadas em hospital de referência por 5 anos (2012–2017). Foi realizada revisão dos prontuários médicos e os resultados são descritos em médias e frequências. A regressão de Poisson foi usada para identificar os fatores independentemente associados à ocorrência de pré-eclâmpsia superajuntada.

**Resultados** Um total de 385 mulheres foram incluídas no presente estudo, e a maioria tinha idade > 35 anos, era multípara, majoritariamente brancas e obesas antes da gravidez. Um terço teve pré-eclâmpsia em gestação anterior, e 17% apresentavam lesão de órgão-alvo associada à hipertensão, majoritariamente disfunção renal. Um total de 85% das pacientes usaram ácido acetilsalicílico e carbonato de cálcio para a profilaxia de pré-eclâmpsia, sendo que a frequência de pré-eclâmpsia superajuntada foi de 40%, com um início prematuro (32.98 ± 6.14 semanas). Destas, 40% apresentaram sinais de gravidade associados à pré-eclâmpsia, com 5 casos de síndrome HELLP; entretanto sem nenhum caso de eclampsia ou morte materna. A incidência de cesárea foi alta, com idade gestacional de 36 semanas ao parto, e um terço dos recém-nascidos tiveram complicações ao nascimento. Um terço das mulheres permaneceu usando medicamentos anti-hipertensivos ao fim da gravidez.

**Conclusão** A hipertensão crônica se relaciona com alta prevalência de pré-eclâmpsia, cesárea, prematuridade e complicações neonatais. Vigilância e cuidado multidisciplinar são importantes para o diagnóstico precoce das complicações.

## Introduction

Chronic hypertension (CH) is one of the most important diseases around the world and is the main risk factor for vascular and neurological complications, which are the first cause of death in high and middle income settings, including Brazil.^1^ ^2^ The American Heart Association 2017 Guideline for Hypertension Detection in Adults considers systolic blood pressure > 130mmHg or diastolic blood pressure > 80mmHg as the first stage of hypertension. Chronic hypertension affects between 3 and 5% of pregnancies and is associated with higher risk of preeclampsia (PE), increased rates of C-sections, placental abruption, prematurity, and perinatal complications. The diagnosis during pregnancy is defined as blood pressure levels > 140 or 90 mmHg prior to conception or before 20 weeks of gestational age.^3^ Women with previous organ dysfunction due to CH have increased risk of adverse maternal and perinatal outcomes.^4^

Pre-eclampsia is diagnosed if hypertension occurs after 20 weeks with significant proteinuria or, in the absence of proteinuria, other organ dysfunctions. Significant proteinuria is defined as total 24-hour urinary proteinuria ≥ 300mg or a spot proteinuria/creatinine rate ratio ≥ 0.26 mg/mg (usually rounded to 0.30 mg/mg) or a dipstick value ≥ 2 + . The organ dysfunctions considered are: hematological complications (thrombocytopenia, with a platelets count ≤ 150,000 mm3, disseminated intravascular coagulation or hemolysis), acute kidney injury (creatinine ≥ 1.1mg/dL), liver involvement (elevated transaminases ≥ 40 IU/L, with or without right upper quadrant or epigastric abdominal pain), or neurological complications (eclampsia, altered mental status, blindness, stroke, clonus, severe headache, and persistent visual scotomata)^3^ Superimposed PE (sPE) can affect between 13 and 50% of the cases, frequently with an early onset disease (before 34 weeks of gestational age).^5^

The pathophysiology of sPE is not only determined by impaired placental trophoblast invasion, but also by vascular and endothelial damage present in CH, with predominance of vasoconstrictors, incomplete remodeling of the uterine artery and greater oxidative stress.^3^ Major complications of hypertensive disorders during pregnancy are HELLP syndrome and eclampsia.^6^ The present study aims to evaluate the maternal and perinatal outcomes of women with CH and factors associated to superimposed PE, followed at a referral center.

## Methods

This is a retrospective cohort study with medical chart review of all women with CH followed at the specialized antenatal care of the Women's Hospital at the Universidade de Campinas (UNICAMP, in the Portuguese acronym), Campinas, state of São Paulo, Brazil, from January 2012 to May 2017. The research was approved by the local ethical board (CAAE: 19451213.2.0000.5404) and informed consent was waived due to data collection from medical charts, in a retrospective approach, with no intervention involved. Women who did not deliver at the considered institution were excluded of maternal and perinatal outcomes. Data on epidemiological and sociodemographic characteristics, previous medical history, obstetrical history, information on time since CH diagnosis and treatment (prior and during pregnancy), data of hospitalization during pregnancy, maternal outcomes, information on delivery and perinatal outcomes were retrieved, and also data on postpartum care (for blood pressure control, use of medication, breastfeeding and contraceptive method). Data was retrieved using a specific Excel (Microsoft Corporation, Redmond, WA, USA) spreadsheet. Results were described as means and frequencies, using Excel 2013 and Epi Info 7.2 (Centers for Disease Control and Prevention, Atlanta, GA, USA). A multivariate analysis was performed using Poisson regression analysis to evaluate the independently association of factors with superimposed sPE (variables selected as predictors were: age, ethnicity, schooling, body mass index (BMI), hypertensive organ damage, diabetes and history of PE in previous pregnancies.

## Results

**Fig. 1:**
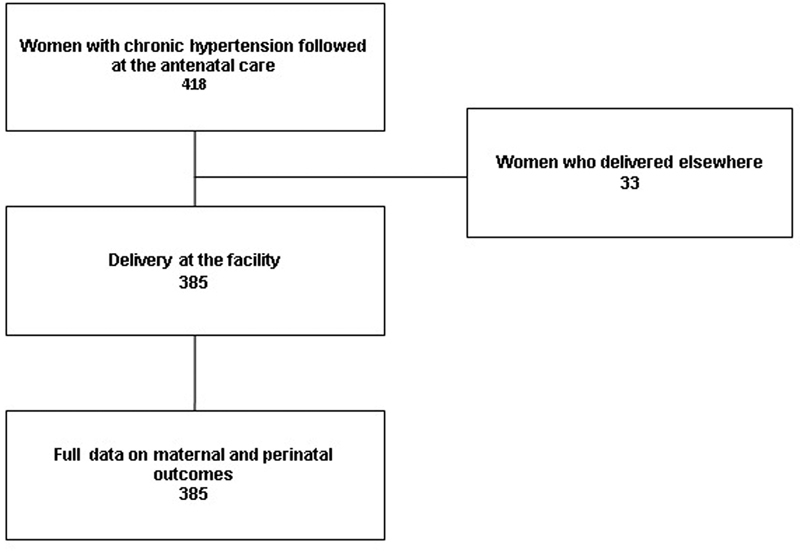
Flowchart of inclusion of women in the present study.

A total of 418 women with CH were identified and were included for sociodemographic analysis; of those, 385 delivered at the facility and their maternal and perinatal results were included.Fig. 1 is the flowchart of the inclusion of cases in the present study.

The mean age of the included women was 31.25 ± 5.96 years old, with a mean of schooling of 9.01 ± 3.16 years. Women were mostly white (65.79%), and obese before pregnancy, with an initial mean BMI of 32.13 ± 9.38 kg/m^2^, and the weight gain during pregnancy was 8.17 ± 10.58 kg. The majority of women had the CH diagnose before pregnancy, but 24.07% were diagnosed during antenatal care, due to hypertension criterion before 20 weeks of gestational age. The most common associated disease was obesity (64.83%), but also diabetes (31.34%) and anemia (26.86%) were frequent (Table 1). The majority of women (77.9%) were multiparous; < 30% of them had a previous preterm birth while more than one third (36.2%) had PE in a previous pregnancy, with a mean gestational age at diagnosis of 33.55 ± 4.16 weeks (Table 1). Among patients with PE history, 91 (28.17%) had childbirth anticipation because of complications associated to PE. Out of the whole cohort, 211 (50.47%) women used antihypertensive drugs before pregnancy, and 80 of them (37.91%) needed association of antihypertensive drugs for adequate blood pressure control. Overall, 65 women (16.88%) presented organ damage associated to hypertension, and kidney dysfunction was the most frequent (Table 1). During pregnancy, 363 women (86.60%) needed antihypertensive drugs for blood pressure control, and of those, the majority (84.68%) had adequate control with only one medication, with no need to increase the dosage until childbirth (Table 1). Pre-eclampsia prophylaxis with aspirin and calcium was prescribed for the majority of women followed (85 and 87%, respectively), and they had a mean number of medical visits > 8. Considering fetal evaluation, fetal growth restriction and Doppler with fetal distress (impaired Doppler) were the most common finding at ultrasound scan.

**Table 1:** Sociodemographic, clinical and obstetrical data of included women

Variables	n(%)
Age (median ± SD) (years old)	31.25 ± 5.96
Years of schooling (mean ± SD)	9.01 ± 3.16
Ethnicity (n.%)	
White	275 (65.79)
Non white	143 (34.21)
Weight before pregnancy (mean ± SD) (kg)	83.17 ± 22.31
BMI before pregnancy (mean ± SD) (kg/m^2)^	32.13 ± 9.38
Gestational Weight gain (mean ± SD) (kg)	8.17 ± 10.58
Obesity n (%)	271 (64.83)
Diabetes n (%)	131 (31.34)
Anemia n (%)	112 (26.86)
Kidney disease* n (%)	35 (8.37)
Hypotyireoidism n (%)	24 (5.74)
Heart Disease** n (%)	23 (5.50)
Primigravida (n.%)	92 (22.01)
Multiparous (n.%)	326 (77.9)
History of Prematurity^1^ (n.%)	
Yes	95 (29.23)
No	224 (8.92)
History of PE in another pregnancy (n.%)	
Yes	118 (36.20)
No	202 (61.96)
Gestational Age at PE diagnosis on previous pregnancy (mean ± SD) (weeks)	33.55 ± 4.16 weeks
History of medically-indicated preterm delivery due to PE complications	
No	46 (38.98)
Yes	72 (61.01)
Previous PE complications	
Fetal/neonatal death	36 (41.86)
Eclampsia	12 (13.95)
Others	7 (8.13)
Placental abruption	7 (8.13)
Fetal malformation	2 (2.32)
Previous diagnosis of chronic hypertension (before pregnancy)	
Yes	274 (75.9)
No	87 (24.1)
Previous use of antihypertensive drugs^3^	
No	179 (45.87)
Yes	211 (50.47)
Previous organ damage due to hypertension	
Hypertensive nephropathy	34 (8.13)
Hypertensive cardiopathy	25 (5.98)
Hypertensive retinopathy	6 (1.43)
Antihypertensive drugs use during pregnancy	
No	55 (13.15)
Yes	363 (86.60)
Need of increasing doses of antyhipertensive drugs	
No	252 (60.28)
Yes	166 (39.71)
Antihypertensive drug association	
No	354 (84.68)
Yes	64 (15.31)
Cardiological evaluation during antenatal care	258 (61.72)
Use of aspirin as PE prophylaxis	356 (85.17)
Gestational age of prescription (mean ± SD)	17.78 ± 5.79 weeks
Gestational age of use interruption (mean ± SD)	33.71 ± 2.64 weeks
Use of calcium carbonate as PE prophylaxis	367 (87.79)
Gestational age at prescription (mean ± SD)	18.06 ± 5.81 weeks
Gestational age at use interruption (mean ± SD)	36.34 ± 3.78 weeks

Abbreviations: BMI, body mass index; PE, preeclampsia; SD, standard deviation.

* Nephrolithyasis; Congenital single kidney; Renal pelvis duplicit.

** Arrhythmia, mitral valve prolapse and coronary disease;^1^ Missing: 6 cases;^2^ Missing: 57 cases;^3^ Missing: 28 cases.

A total of 41.14% of the total pregnancies with CH had sPE, mainly with early onset (32.98 ± 6.14 weeks of pregnancy), with 77 cases (44.76%) with severe features of PE; however, no cases of eclampsia or maternal death were reported in this cohort (Table 2). Severe hypertension and headache were the most frequent symptoms presented by those women. All of the women with severe features of PE were managed with magnesium sulfate to prevent seizures.

**Table 2:** Diagnosis and outcomes of women with superimposed preeclampsia among chronic hypertension

Variable	*n* (%)
Superimposed preeclampsia	172 (44.67)
Gestational age at diagnosis (mean ± SD) (weeks)	32.98 ± 6.14 weeks
Postpartum diagnosis	12 (6.97)
Preeclampsia with severe features	77 (44.76)
Magnesium sulfate use	77 (100)
Need for increased maintenance dose	11 (14.28)
> 1 Zuspan scheme	23 (29.87)
Drug administration time (Mean ± SD) (hours)	42.33 ± 21.45
Most frequent findings among women with severe features	
Severe hypertension*	71 (42.77)
Headache	56 (33.73)
Visual symptoms	23 (13.85)
Epigastric pain	16 (9.63)
Pre-eclampsia without proteinuria at diagnosis (< 300 mg/24 hour)	36 (20.93)
HELLP syndrome	5 (1.29)

Abbreviations: SD, standard deviation.

We observed a high incidence of C-sections (69.1%) mainly due to “worsening of maternal condition” (22.01%), and also because of a history of repeated C-sections (≥2 previous cesareans) (21.26%). The mean number of days of hospitalization during pregnancy was 7.59 ± 5.66, including admission for childbirth (Table 3). Loss to follow-up after delivery was high and only 48% of the women returned to a postpartum visit at the facility; however, among them, the majority was breastfeeding and received a prescription for contraception.

**Table 3:** Childbirth, hospitalization and postpartum data of included cases (*n* = 385)

Variables	*n* (%)
Mode of delivery	
Vaginal delivery	117 (30.3)
Cesarean section	268 (69.1)
Indications	
Maternal condition	59 (22.01)
Repeated c-section	57 (21.26)
Fetal distress	45 (16.79)
Failed induction of labor	32 (11.94)
Cephalio-pelvic desproportion	21 (7.83)
Gestational age at birth (mean ± SD) (weeks)	36.73 ± 3.077
Number of hospitalizations for blood pressure control	1,66
Days of hospitalization during pregnancy (mean ± SD)	7.59 ± 5.66
Prescription of antihypertensive drugs after hospital discharge	195 (50.64)
Postpartum medical visit at Women's Hospital	188 (48.33)
Exclusive breastfeeding at postpartum visit	105 (55.85)
Antihypertensive use at postpartum visit	128 (33.24)
Use of contraceptive method	117 (62.23)

Abbreviations: SD, standard deviation.

The mean gestational age at birth was 36.73 ± 3.07 weeks, with a majority of 5^th^ minute Apgar score ≥ 7 (> 85% of cases). However, 29.87% (115) of the newborns presented complications such as respiratory distress, jaundice or low weight at birth, demanding admission to the neonatal intensive care unit (Table 4).

**Table 4:** Perinatal outcomes of women with chronic hypertension (*n* = 385)

Variables	n(%)
Birthweight (mean ± SD)	2995.90 ± 718.26
Capurro (mean ± SD)	37.77 ± 1.95
Newborn weight adequacy for gestational age	293(76.10)
5th minute Apgar score < 7	24(6.23)
Neonatal complications*	115(29.87)

Abbreviations: SD, standard deviation.

After medical discharge, all women were scheduled for a postpartum evaluation (6 weeks postpartum), however, only 188 women showed up (48.33%), and of those, 128 (33.24%) were using antihypertensive drugs at this evaluation and 62.23% were using contraception (Table 3). Considering the high rates of superimposed PE, we performed a multivariate analysis to evaluate if there were factors independently associated with this outcome. The Poisson regression analysis showed that none of the factors studied had statistical significant association with sPE (Table 5).

**Table 5:** Poisson regression analysis for factors associated to developing superimposed preeclampsia (*n* = 418)

Variables	Categories	*p-value*	P.R.*	95%CI PR
Age (years old)	< 35 years	—-	1,00	—
	≥ 35 years	0.834	1.04	0.75–1.42
Ethnicity	White	—	1.00	—
	Non-white	0.852	1.03	0.75–1.41
Schooling	< 11 years	—	1.00	—
	≥ 11 years	0.759	0.95	0.71–1.29
Body Mass Index	< 25 kg/m^2^	—	1.00	—
	≥ 25 kg/m^2^	0.203	0.81	0.58–1.12
Nephropathy due to hypertension	No	—	1.00	—
	Yes	0.402	1.24	0.75–2.04
Retinopathy due to hypertension	No	—	1.00	—
	Yes	0.331	1.64	0.61–4.41
Cardiopathy due to hypertension	No	—	1.00	—
	Yes	0.921	0.97	0.51–1.83
Diabetes	No	—	1.00	—
	Yes	0.882	0.98	0.71–1.35
Previous pre-eclampsia	No	—	1.00	—
	Yes	0.265	1.20	0.87–1.65

Abbreviations: CI, confidence interval; PR, prevalence ratio.

## Discussion

Our study showed a high incidence of sPE among women with CH followed at a referral center. Those women received prophylaxis for PE and were followed by a multidisciplinary team. Preterm delivery was an important perinatal complication in our cohort and loss to follow-up after delivery affected half of the women in this cohort. Also, C-section rates were high in this specific group of women.

Almost all of the women were > 30 years old; prevalence of CH is higher in individuals > 25 years old, affecting 10.5% of women with ages between 25 and 34 years old and 19.5% after 35 years old in Brazil.^7^ Obesity was also very frequent, and the relationship between this condition and CH is well recognized: Hubert et al^8^ showed that 61% of the women with CH had the disease attributed to obesity.

Normal weight gain during pregnancy among women with BMI > 30kg/m^2^ is 0.17 to 0.27 kg/week. If we consider that the mean gestational age at birth was ∼ 37 weeks, the estimated normal weight gain would be from 6.29 to 9.90 kg.^9^ The mean weight gain in our cohort was 8.17kg. Adequate weight control was achieved due to a specialized and multidisciplinary antenatal care, with nutritional support and orientation.

The majority of women had the diagnosis of CH before pregnancy; however, around a quarter of them received this diagnosis during antenatal care, because of high blood pressure levels prior to 20 weeks of gestation. Underlying clinical conditions are often not diagnosed prior to gestation. Studies showed that only 15% of the patients reported preconceptional healthy behavior, and antenatal care is the opportunity to investigate, diagnose and treat, if necessary, chronic diseases, improving maternal and perinatal outcomes during that pregnancy but also promoting long-term effects on the health of the women.^10^

A lot of interventions have to be performed among CH pregnant women to avoid maternal and perinatal adverse outcomes, such as adequate blood pressure control, fetal vitality evaluation and screening of organ damage. However, there is still great discussion on when to start medication. Overall, it is started in women with blood pressure levels > 150/100 mm Hg, and a lot of drug classes may be safely prescribed.^11^ In Brazil, the most used and available is methyldopa. Although almost 50% of the women did not use antihypertensive drugs before pregnancy, during antenatal care, > 80% of them needed such intervention. On the other hand, < 20% of them had to associate two or more classes of antihypertensive drugs for adequate blood pressure control.

Aspirin and calcium carbonate are widely studied as PE prophylaxis,^3^ and their prescription is part of the protocol of the institution during antenatal care of women with CH. Calcium supplementation among women with low dietary intake has demonstrated significant impact in reducing PE.^3^

Extensive research has supported the use of low dose aspirin, specially prior to 16 weeks of pregnancy for PE prophylaxis,^3^ however, specific analysis for the subgroup of CH women in the ASPRE trial^12^ has questioned the efficacy of such intervention, reasoning that this population has other baseline changes related with the physiopathology of hypertension, mainly vascular and endothelial, that can cause, by themselves, increased risk for sPE, not related with placental deficit. However, due to the low risk of potential complications associated to the use of this drug, we support the prescription of low dose aspirin to women with CH.

A previous report showed that ∼ 11% of women with CH had proteinuria prior or in the beginning of pregnancy, caused by nephrosclerosis or, less frequently, undiagnosed chronic kidney disease.^13^ In our study, 8.13% of the women with CH had kidney damage, and 12.98% had proteinuria at the initial laboratorial investigation. The risk of sPE is higher in patients with underlying renal disease.^14^ Also, superimposed PE can affect the kidney functionally and morphologically during pregnancy, and can cause renal damage after childbirth.^14^ Furthermore, microalbuminuria is related with renal vascular damage and systemic endothelial dysfunction, and has been associated with cardiovascular events.^14^

The overall prevalence of sPE varies greatly in the literature, from 13 to 50% of the cases.^3^ Our prevalence of sPE was 44.67%, similar to a study in the Asian population (with 43.3% of SPE).^15^ However, in the aforementioned study, less than half had PE with severe features. In our sample, we acknowledge that there might be a selection bias, once cases considered of higher risk among CH are the ones referred to our antenatal care, and those are most likely at increased risk of sPE and severity.

All of the cases with severe features used magnesium sulfate, a known intervention to prevent eclampsia.^3^ Furthermore, no cases of eclampsia or maternal death were reported in our sample, probably due to close surveillance and identification of complications and adequate decision on timing of delivery.

We found a high rate of C-sections, ∼ 70%, specified in the medical chart as due to maternal complications. Cesarean section rates are increasing worldwide, and Brazil is one of the leaders, with an overall prevalence of 51.9%, while in the facility the global C-section rate is ∼ 40%.

Fetal assessment was performed by clinical evaluation (fundal height measurements), cardiotocography (weekly after 28 weeks) and ultrasound/Doppler study (monthly or more frequent when abnormal previous exam or clinical evaluation were observed). Fetal growth restriction (FGR) was the most frequent ultrasound finding (considered when estimated fetal weight was below the 10th centiles^5^), which is in accordance with other studies.^15^

Almost all of the women who received antihypertensive prescription at medical discharge returned for postpartum care at our hospital, but only 33% of them were still using it, because of adequate blood pressure control at home (checked at the postpartum evaluation). During the postpartum visit, there is a clinical and gynecological evaluation, with contraception orientation. A total of 62.23% of the patients were already using contraception, mostly surgical sterilization (94.01%), because the majority were multiparous and were eligible by the Brazilian legislation for this procedure. Women with no established contraceptive method at birth received this orientation at the postpartum visit.

Evaluation of long-term cardiovascular risk is important during the postpartum visit, especially in women with sPE, because endothelial dysfunction is a predictor of coronary events.^16^ ^17^ Also, the persistence of recognized markers of cardiovascular disease can be observed after childbirth in women with sPE, like high serum uric acid levels and microalbuminuria.^16^ It is estimated that the relative risk of cardiovascular disease in women with hypertensive disorders in pregnancy varies between 2.3 to 3.7 times more than in the baseline population, and the risk of venous thromboembolic events and hemorrhagic stroke is also higher.^17^

Our study has several limitations: the small number of women followed; the methodological approach of medical chart review; a possible bias of selection since most severe cases of CH are referred quickly to specialized care, while mild CH cases are conducted elsewhere; and follow-up at a hospital with more resources allows women to receive better treatments, which is not true in the majority of facilities in Brazil. Even with these limitations, we believe our findings can be considered and we strongly support that a multidisciplinary approach, with a team composed by obstetrician, cardiologist, nutritionist, physical educator and others, performing a close surveillance for women with CH is the key for good outcomes.

## Conclusion

Our study showed that CH during pregnancy impacts maternal and perinatal outcomes, with high prevalence of sPE, use of antihypertensive drugs, C-sections and neonatal complications. A specialized antenatal care is fundamental to provide better maternal and perinatal outcomes, through close follow-up and early diagnosis of complications. Postpartum care is frequently underestimated and should be the focus of future interventions to allow for better long term assessment of complications and contraceptive counseling.
